# Effect of maize root exudates on indole-3-acetic acid production by rice endophytic bacteria under influence of L-tryptophan

**DOI:** 10.12688/f1000research.13644.1

**Published:** 2018-01-25

**Authors:** Arun Karnwal, Aradhana Dohroo

**Affiliations:** 1 School of Bioengineering and Biosciences, Lovely Professional University, Jalandhar, Punjab, India; 2 Bhojia Institute Of Life Sciences, Budh (Baddi), Solan, Himachal Pradesh, India

**Keywords:** Pseudomonas fluorescens, endophytes, maize, rice, tryptophan

## Abstract

**Background**: It is assumed that plant growth regulators produced by beneficial bacterial species could also influence plant growth. IAA is a major plant growth regulator responsible for stimulation of plant growth. There are several microorganisms which are naturally responsible for L- tryptophan metabolism.

**Methods**: In total, 56 indigenous morphologically distinct isolates from rice roots were selected and subsequently characterized with biochemical tests, 16S rRNA sequencing and plant growth promoting activities. *Pseudomonas* *fluorescens* RE1 (GenBank: MF102882.1) and RE17 (GenBank: MF103672.1) endophytes resulted in better PGP activity against the other 54 isolates. Both endophytes were tested to screen indole-3-acetic acid production ability in pure culture conditions with L-tryptophan at 0, 50, 100, 200 and 500µg/ml concentrations.

**Results**: *P.* *fluorescens *RE1 was recorded efficient for indole production in comparison to *P. fluorescens* RE17 at various L-tryptophan concentrations. *P. fluorescens* RE1 was shown to produce between 0.8 µg/ml and 11.5µg/ml of indole at various tryptophan concentrations, while RE17 produced between 1.2µg/ml and 10.2µg/ml. At 200 and 500µg/ml tryptophan concentration, *P. fluorescens* RE17 produced 7.4pmol/ml and 9.3pmol/ml IAA, respectively.

**Conclusions**: Inoculation of maize seed with *P. fluorescens* RE1 and RE17 showed a significantly higher level of IAA production in comparison to non-inoculated seeds. Current study outcomes proved that plant growth regulators produced by Pseudomonas species could also play a critical role in plant growth promotion.

## Introduction

Indole-3-acetic acid (IAA) is one of the most physiologically active phytohormones, and is a product of L-tryptophan metabolism ( Yu *et al.*, 2017). 80% of rhizospheric microorganisms naturally yield auxins as secondary metabolites due to the rich supplies of root exudates ( Myresiotis *et al.*, 2015). The rhizosphere is a highly selective area for host-microbe interaction, and during their life span few microbes may enter inside the plant tissue and stay without causing any negative symptoms ( Santoyo *et al.*, 2016). These indigenous colonizers reside in almost all internal tissues/cells of plant ranging from tissues of the roots to stem, leaf, flower, fruit and seed ( Souza *et al.*, 2015).

These endophytes actively or passively facilitate modification of morphology in the plant cell. It is reported by many workers that endophytes promote growth due to favourable adaptations to abiotic or biotic stresses ( Nutaratat *et al.*, 2014). Indigenous bacteria also promote plant growth through various mechanisms. These include direct and indirect mechanisms. Direct mechanisms involve various plant growth promoting hormones (i.e. indole-3-acetic acid, gibberelic acid, adenine-type cytokinins, phenylurea-type cytokinins, ethylene, and abscisic acid), solubilizing inorganic phosphate and atmospheric N _2_ fixation. However, indirect mechanisms involve production of various antimicrobial chemical, siderophores and lytic enzymes against the plant pathogens ( Zhang *et al.*, 2013). The aim of the present study is to analyse the effect of L-tryptophan and maize root exudates on the production of IAA by rice endophytic bacteria.

## Methods

### Isolation and characterization of endophytes

A total of forty healthy rice ( *Oryza sativa* L. basmati **)** plants were randomly selected and collected from agricultural land situated at Dehradun (30° 19’ N, 78° 04’ E) Uttarakhand, India. Surface-sterilized roots were dissected into small pieces and 1g fresh root tissue was ground in sterile mortar and pestle with 0.85% sterilized saline solution. The ground tissue extract was serially diluted (sevenfold) in sterile saline and 100 µL aliquots were spread on nutrient agar plates (Hi-Media, India). Biochemical characterization of 56 endophytic isolates was carried out as described in Bergey’s manual of determinative bacteriology ( Holt *et al.*, 1994). On the basis of higher indole production ability, two isolates RE1 and RE17 were selected for further study and 16S rRNA analysis.

### 16S rRNA sequencing

16S rRNA sequencing and phylogenetic analysis were done for both isolates RE1 and RE17. Universal 16S rRNA primers (8F 5’ AGAGTTTGATCCTGGCTCAG 3’ and U1517R 5’ ACGG(A/C)TACCTTGTTACGACTT 3’) were used for 16S rRNA amplification of bacterial isolates under PCR reaction ( Puente *et al.*, 2004). The ProbeBase online utility was used to check primers specificity and the BLAST search facility at the NCBI (Genbank database). Multiple sequence alignment was performed by using MUSCLE alignment algorithm and PhyML software was applied for phylogenetic analysis ( Dereeper *et al.*, 2008).

### Indoles and indole-3-acetic acid (IAA) production

Isolate RE1 and RE17 were tested for indole production by using the method described by Karnwal (2009). Indole production was analyzed by mixing 4ml of Salkwaski’s reagent in 1ml of cell free filtrate. This mixture was incubated at 28 ±2°C for 15 min to observe pink coloration as positive indication of IAA production. Absorbance was measured at 535 nm using UV-VIS Spectrophotometer 2201 (Systronics, India). Standard curve of IAA was used to quantify indole production by bacterial isolates ( Karnwal, 2009). ELISA (Phytodetek, Agdia Inc, Elkhart, IN, USA) was used to estimate IAA produced by RE1 and RE17 as described by Karnwal (2009).

### IAA production in growth pouch with maize seeds

Maize ( *Zea mays* L. Kissan) seeds were procured from the local market of Dehradun (30° 19’ N, 78° 04’ E) Uttarakhand, India. Surface sterilized seeds were soaked in 10 ml of bacterial suspension, grown in half strength tryptic soy broth (TSB), until 10 ^8^ cell density was achieved after gentle agitation for 10–15 min. Pre-sterilized growth pouches supplemented with 30ml ml of sterile half-strength N-free Hoagland’s nutrient solution were inoculated with bacterial treated seeds aseptically (3 seeds per pouch and 3 pouches per treatment). Seeds treated with 0.1 M MgSO _4_ were considered as controls.

Bacteria layered maize seeds were inoculated in growth pouches and cultivated inside plant growth chambers at 100rpm. IAA concentration from the growth pouch supernatant was determined with ELISA (Phytodetek, Agdia Inc, Elkhart, IN, USA). Stock solutions of the IAA (10 µmole/ml) were prepared within absolute methanol and standard concentrations 78–2500 pmoles/ml (IAA) were used for standard curve preparation.

## Results

**Figure 1.**
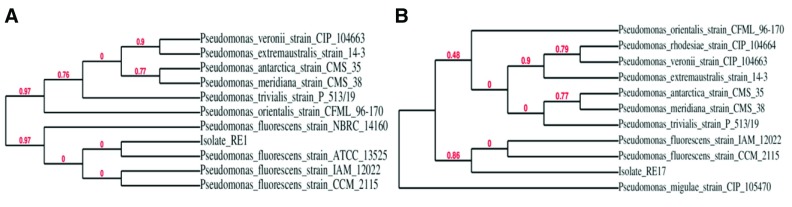
Phylogenetic tree of bacterial isolates created by using TreeDyn, Tree Rendering software based on MUSCLE alignment data. ( **A**) BLAST similarity search results and phylogenetic tree for isolate RE1; ( **B**) Phylogenetic tree for isolate RE17.

In the present study, 5.6 × 10 ^1^ CFU/ml morphologically unambiguous indigenous bacteria were purified and selected for further analysis for phytohormone IAA production ability. Isolates RE1 and RE17 were identified on the basis of Gram stain, biochemical activities and sugar fermentation, as described in Bergey’s manual of determinative bacteriology ( Holt *et al.*, 1994) ( Table 1). BLAST analysis of the 16S rRNA gene sequence of RE1 and RE17 isolates demonstrated maximum sequence similarity with the *P. fluorescens* strain ATCC 13525 (99%, Genbank Sequence ID: NR_114476.1) and *P. fluorescens* strain CCM 2115 (98%, Genbank Sequence ID: NR_115715.1), respectively as shown in phylogenetic tree analysis by using MUSCLE algorithm and PhyML phylogenetic tree creation software ( Figure 1).

**Table 1.** Biochemical characteristics of *Pseudomonas* isolates.

	Test microorganism	
Tests	RE1	RE17
Gram staining	G-ve	G-ve
Pigmentation (on King’s B medium)	+	+
Fluorescence under UV light	+	+
Starch hydrolysis	-	+
Citrate utilization	+	+
Oxidation reaction	+	+
Casein hydrolysis	+	+
Urease production	+	+
Catalase test	+	+
Gelatinase production	-	+
Indole production	+	+
Siderophore production	+	-
H _2_S production	+	+
Glucose	=	+
Mannitol	+	+
Fructose	+	+
Arabinose	+	-
Trehalose	+	-
Glycerol	+	-
Xylose	+	+
3-ketolactse production	+	+

**Figure 2.**
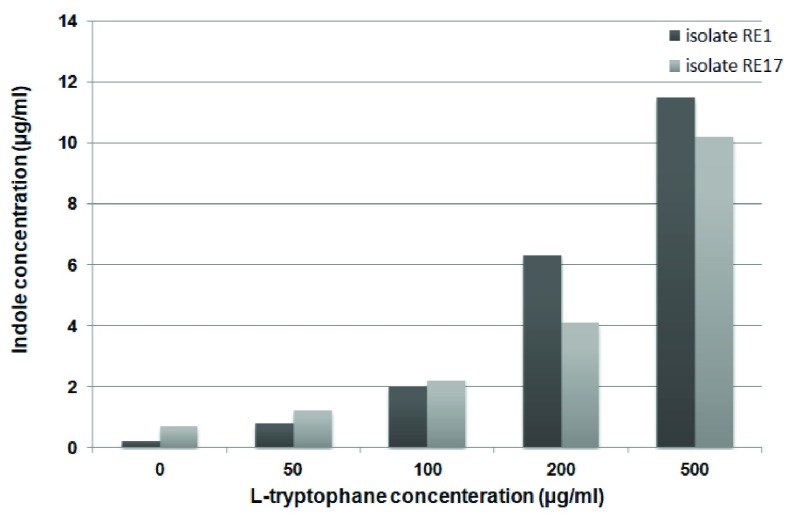
Production of indoles (µg/ml) by RE1 and RE17 at various concentrations of L-tryptophan.

**Figure 3.**
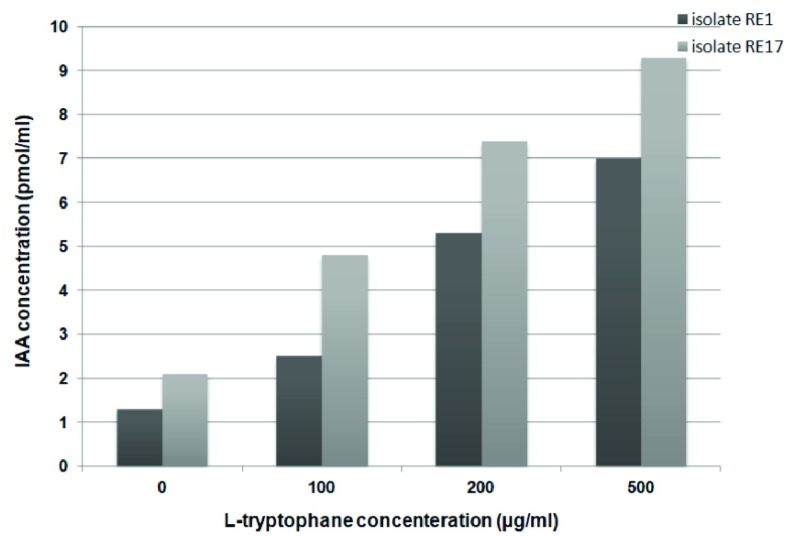
Production of IAA (pmol/ml) by RE1 and RE17 at various concentrations of L-tryptophan

In the deficiency of L-tryptophan, strain RE17 released considerable levels of indole (0.7µg/.ml) in comparison with RE1 (0.2µg/ml). In the presence of 50µg/ml of L-tryptophan, RE17 produced significantly higher concentrations of indole compared to RE1 ( Figure 2). When 200µg/ml of L-tryptophan was added to the medium, RE1 and RE17 produced eight, and three times the concentration of indole produced at 50µg/ml of L-tryptophan concentration, respectively ( Figure 2). It has been observed that RE17 has greater IAA production ability than RE1 ( Figure 3). Varying levels of IAA were recorded with different concentrations of tryptophan, i.e. 0, 100, 200 and 500 μg/ml by RE1 as shown in Figure 3.

**Figure 4.**
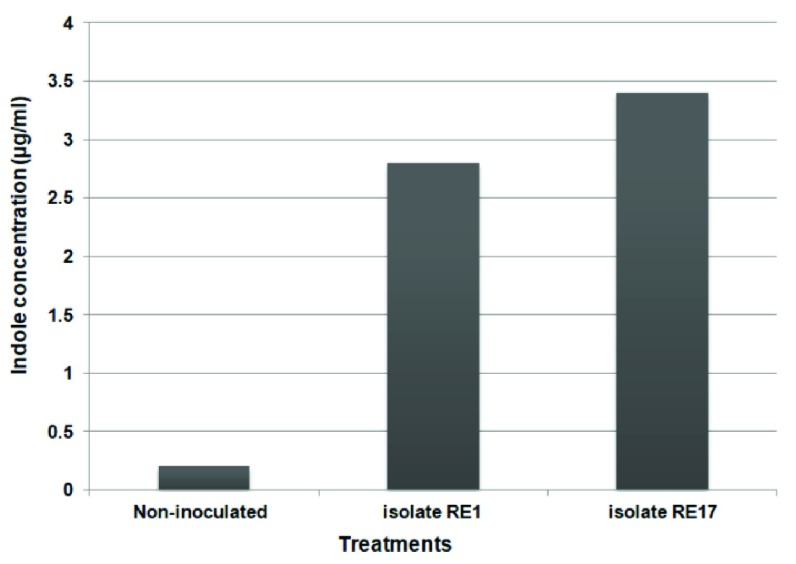
IAA concentration in growth pouches inoculated with or without indigenous isolates.

The concentrations of IAA secreted with maize root exudates in growth chamber studies with RE1 and RE17 were significantly higher (2.8 pmol/ml and 3.4 pmol/ml) than that of the control (0.2 pmol/ml), as shown in Figure 4.

## Discussion

Indigenous microbes colonize internal regions of the plant and are present in almost every plant globally ( Ji *et al.*, 2010). IAA secretion through endophytes is a beneficial trait leading to plant development ( Ahmad *et al.*, 2008). Therefore, current studies examine IAA generating endophytic *P. fluorescens* strains from rice roots, with many reporting the active role of tryptophan in the production of IAA by plant growth promoting bacteria ( Karnwal, 2017; Stachecki *et al.*, 2004). Tryptophan improves IAA biosynthesis in *P. fluorescens* strains associated with current research, revealing that it might be the precursor for IAA biosynthesis in these bacterial strains ( Khan & Bano, 2016). It has been shown that L-tryptophan concentration affects biosynthesis of IAA at significant levels ( Ignatova *et al.*, 2015), however, in this study IAA production was between 1.3 and 9.3pmol/ml in both *P. fluorescens* strains used, with or without tryptophan. Using ELISA based studies, it was revealed that strain RE17 was the best IAA producer in the presence of maize root exudates in growth chamber study.

The rhizosphere is a rich environment for growth of microorganisms, and it generates a great diversity of microbes ( Balseiro-Romero *et al.*, 2016; Kierul *et al.*, 2015; Vaishnav *et al.*, 2016). The growth chamber study confirmed the beneficial potential of maize root exudates on IAA secretion. This supports the fact that plant roots produce some chemical substances (root exudates) that support the growth of rhizospheric microorganisms and their colonization ( Kierul *et al.*, 2015; Pereira & Castro, 2014). Hence, these strains have a potential of being developed as bio-inoculants.

## Data availability

**Bacterial isolate sequence data at NCBI:**

Pseudomonas fluorescens strain RE1 16S ribosomal RNA gene, partial sequence: GenBank: MF102882.1.

Pseudomonas fluorescens strain RE17 16S ribosomal RNA gene, partial sequence: GenBank: MF103672.1.

**Raw data for biochemical analyses and IAA production:** http://doi.org/10.17605/OSF.IO/X5Q46 ( Karnwal, 2018).
