# Supplementary material for: Effect of maize root exudates on indole-3-acetic acid production by rice endophytic bacteria under influence of L-tryptophan
Source: F1000Res. 2018 Jan 25;7:112. [Version 1] doi: 10.12688/f1000research.13644.1 (PMC5829522; doi:10.12688/f1000research.13644.1)
Supplement: Peer review history

# Peer review: Referee response for version 1

**DOI:** 10.5256/f1000research.14823.r30813

**Published:** 2018-02-27

**Authors:** Loganathan Karthik

**Affiliations:** 1 Drug Discovery Laboratory, State Key Laboratory of Bioreactor Engineering, East China University of Science and Technology, Shanghai, China

The topic and work bought into notice are very interesting. The plant growth regulators produced by microorganisms, which could play a critical role in plant growth promotion for the promotion of sustainable agricultural practices.

 The Abstract is well written and appropriate as per work done.

 The Introduction section is well written.

 All Results seems appropriate and is supported by straightforward tables and figures.

 The Discussion is well presented and the findings were evaluated with current articles.

 Minor corrections:

- In Abstract, Conclusions must be change in to Conclusion
- In Introduction part, the following sentence must be reframed - 80% of rhizospheric microorganisms naturally yield auxins as secondary metabolites due to the rich supplies of root exudates
- In results section:
  - Fig 1- the scale bar is missing
  - Fig 2,3,4-  Remove the grid lines
  - Authors must carry out statistics for all results
- Results and discussion part must be combined
- Conclusion part is missing

The article can be indexed in F1000 journal with minor corrections as suggested.

I have read this submission. I believe that I have an appropriate level of expertise to confirm that it is of an acceptable scientific standard.

# Peer review: Referee response for version 1

**DOI:** 10.5256/f1000research.14823.r30252

**Published:** 2018-01-31

**Authors:** Vishal Kumar Deshwal

**Affiliations:** 1 Department of Microbiology, Doon (P.G.) Paramedical College, Dehradun, India

This article addressed to the effect of L-tryptophan and root exudates as inducer for the production of auxin, phytohormone by inoculated bacteria. Many works have been completed with PGP rhizospheric bacteria but naturally occurring indigenous bacteria can provide a better results in comparison to rhizospheric bacteria. In present article:

 Introduction

- This section of article is written in constructive manner to fulfil and provide the basic idea behind the use of bacteria for plant growth and phytohormone.
- It also summarize the role of endophytic bacteria for betterment of plants through various mechanism those are helpful for agriculture.

Methods

- Author’s used basic (biochemical analysis) as well as molecular methods for the characterization of isolates that confirm the authenticity of identification of bacterial spp.
- Author mentioned the proper site of isolation with given altitudes so in future said work can be replicated with samples.
- All methods are explained properly with size of samples and other chemicals that satisfy the requirements if any one likes to duplicate the work in future.

Result

 Characterization and phytohormone production results displayed by author are acceptable. Authors tried to evaluate the effect of endogenous bacteria, isolated from rice, on maize as well as effect of maize root exudates on bacteria and its colonization. Present article is a preliminary stage for the future prospects of isolated bacterial isolates as biofertilizer. Authors have to go further to test both isolates in field condition to use these endogenous isolates as biofertilizer.

 Suggestion:

 Author may add statistical analysis in Figure 2, 3 and 4 so results will show the significance of analysis.

I have read this submission. I believe that I have an appropriate level of expertise to confirm that it is of an acceptable scientific standard.
